# Supplementary material for: A Novel Approach to Deliver Therapeutic Extracellular Vesicles Directly into the Mouse Kidney via Its Arterial Blood Supply
Source: Cells. 2020 Apr 10;9(4):937. doi: 10.3390/cells9040937 (PMC7226986; doi:10.3390/cells9040937)
Supplement: Supplementary file 1 [file cells-09-00937-s001.pdf]

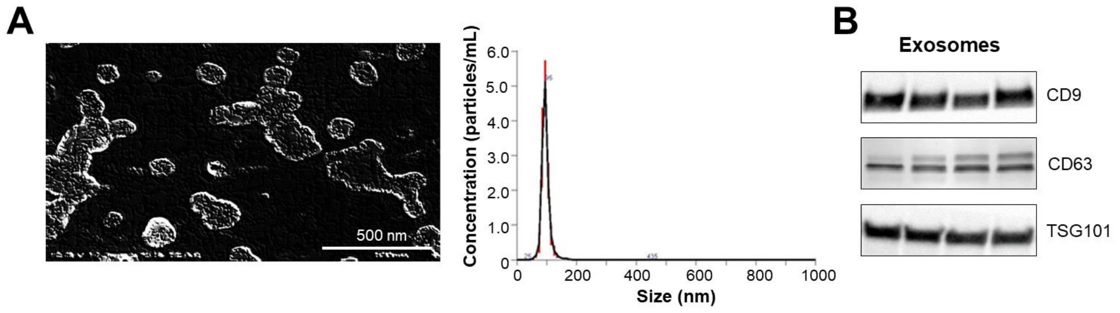

Supplementary Figure 1: Exosome Characterization

(A) Transmission electron microscopy (TEM) of exosomes, and distribution of exosome size measured by nanoparticle tracking analysis. Scale bar represents 500 nm. (B) Validation of exosome surface markers CD9, CD63, and TSG101 by Western blot analysis.
